# The Aging of Adipocytes Increases Expression of Pro-Inflammatory Cytokines Chronologically

**DOI:** 10.3390/metabo11050292

**Published:** 2021-05-01

**Authors:** Bulbul Ahmed, Hongwei Si

**Affiliations:** Department of Human Sciences, Tennessee State University, Nashville, TN 37209, USA

**Keywords:** adipocytes, aging, NF-κB, pro-inflammation

## Abstract

Adipose tissue is a significant producer of pro-inflammatory cytokines in obese and old individuals. However, there is no direct evidence of whether and how aged adipocytes enhance the production of pro-inflammatory markers. We aimed to investigate whether the aging adipocytes increase pro-inflammatory markers. Swiss mouse embryonic-tissue-derived 3T3-L1 cells were differentiated into adipocytes and maintained for 60 days in the conditioned medium or 35 days in the unconditioned medium. Additionally, 20-month-old male C57BL/6 mice were fed a standard chow diet for 37 weeks until they were extremely aged, when ~75% of mice died because of aging. Accumulated lipids, pro-inflammatory markers, and nuclear factor kappa B (NF-κB) pathway markers from differentiated adipocytes were analyzed. Pro-inflammatory markers and NF-κB pathway markers of epididymal white adipose tissues (EWATs) and adipocytes from EWATs were also analyzed. We found that the aging adipocytes chronologically accumulated lipids and increased pro-inflammatory markers interleukin-6 (IL-6), monocyte chemoattractant protein-1 (MCP-1), and tumor necrosis factor-alpha (TNF-α); at the same time, NF-κB p50 markers were also increased while IκBα protein was decreased significantly in conditioned medium. Similar results were observed when differentiated adipocytes were maintained in the unconditioned medium and the adipocytes from EWATs of aged mice. We demonstrated that aging augmented chronic inflammation through the NF-κB signaling pathway in adipocytes and adipose tissue.

## 1. Introduction

Adipose tissue is a large and dynamic endocrine organ responsible for energy storage, making up from 2% to 70% of human body weight [1]. It has the functions of immune modulation, wound healing, and tissue regeneration. The aging of adipose tissues is associated with declined tissue functions and increased disease burden resulting from changes in structural function, cellular composition, and endocrine signaling, while promoting insulin resistance, metabolic dysfunction, chronic inflammation, and impaired regenerative capacity [2]. Besides other age-related chronic diseases, the aging of adipose tissues increases the risk of cancer deaths, which is not only due to hormonal dysregulation alone, but other age-related complications as well [3].

The aging of adipose tissues is frequently associated with increased fat mass and circulating pro-inflammatory cytokines, including tumor necrosis factor-alpha (TNF-α), monocyte chemoattractant protein-1 (MCP-1), and interleukin-6 (IL-6) [4,5]. Chronic pro-inflammation activity is associated with metabolic dysfunctions and insulin resistance, ultimately developing multiple diseases, for example, type 2 diabetes and Alzheimer’s disease [6]. Aging is associated with declining activity and reducing pro-inflammatory cytokine production in the macrophages of peripheral and visceral adipose tissues [7,8], whereas the pro-inflammatory expressions in the stromal vascular fractions (SVCs) have not been observed to change, except IL-6 [7,9]. Some studies demonstrated that the major contributors of adipose tissue inflammation in aging are the occupant adipose tissue immune cells, not the adipocytes [9,10]. However, the differentiated adipocytes from SVCs suggest that the expression of pro-inflammatory markers increase in aged individuals compared to young ones [11,12,13], indicating that the aging of adipocytes in adipose tissue may be responsible for the increasing pro-inflammation.

The cellular and molecular mechanisms of adipose tissue—mainly increased pro-inflammatory cytokine production in aged adipose tissue—remain unresolved. There is no direct evidence of which types of cells are responsible for the increase. We hypothesized that aging adipocytes are responsible for the increasing pro-inflammatory cytokine production in adipose tissues, which eventually causes age-related complications. Therefore, we differentiated 3T3-L1 preadipocytes for an extended period, maintained them for 60 days in conditioned medium (only half of the medium was replaced every two days), and monitored the changes in lipid accumulation, pro-inflammatory cytokine production, and NF-κB pathway markers. Since the medium in our first study was conditioned, which was different from a previous study [14], we further compared the effects of the unconditioned medium (all medium was replaced every two days) on pro-inflammatory markers until day 35. We also fed a chow diet to 20-month-old mice for 37 weeks to observe the changes of the pro-inflammatory markers in visceral adipose tissue, particularly epididymal white adipose tissues (EWATs). Our study demonstrates that the aging adipocytes chronologically induce the production of pro-inflammatory cytokines, accompanied by the enhancement of several key molecules of the NF-κB pathway.

## 2. Results

### 2.1. Lipid Accumulation and Pro-Inflammatory Markers Were Chronologically Increased in Aging Adipocytes during the 60 Days in Differentiated 3T3-L1 Cells

**Figure 1:**
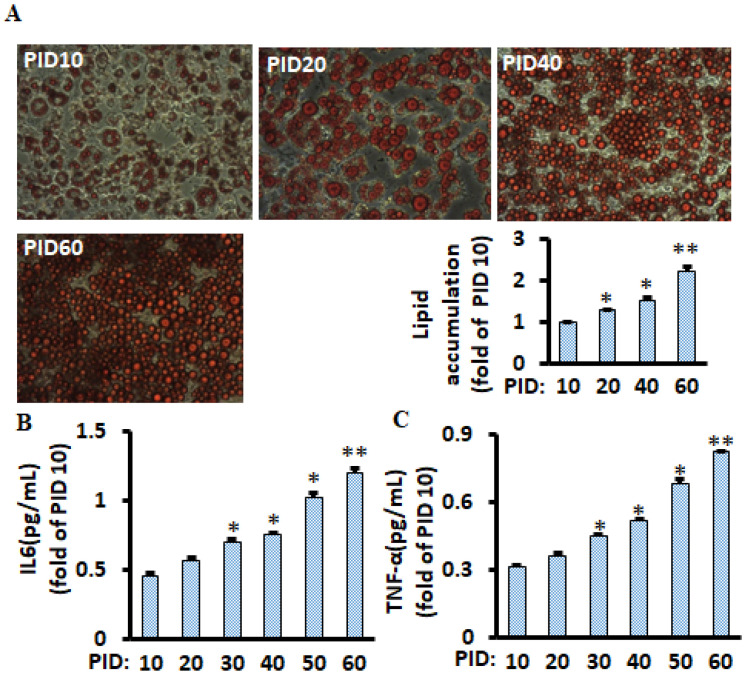
Lipid accumulation and pro-inflammatory markers chronologically increased during the 60 days in differentiated 3T3-L1 adipocytes with conditioned medium. Representative images of lipid accumulation (Oil Red O staining) at relevant days are shown (**A**), and lipids were measured using a spectrophotometer, and a bar graph is shown. Pro-inflammatory markers IL-6 (**B**) and TNF-α (**C**) of differentiated adipocytes were measured using the ELISA kit. Data were analyzed using one-way ANOVA, and significant differences were further analyzed using Tukey’s test; * *p* < 0.05, ** *p* < 0.01 compared to PID 10.

To mimic the physiological microenvironment of fat tissues (continuously producing pro-inflammatory markers and various bioactive molecules), half of the medium (1 mL per well) was replaced every two days in differentiated 3T3-L1 adipocytes throughout the 60 days experimental period. We found that lipid accumulation (Figure 1A) and pro-inflammatory cytokines TNF-α and IL-6 (Figure 1B,C) were continuously increased in the cells. These results are consistent with a previous study that lipids were chronologically accumulated, although the most prolonged treatment was only at post-induction day (PID) 20 in the study [15].

### 2.2. The NF-κB Pathway of Aging Adipocytes Was Increased Chronologically during the 60 Days in Differentiated 3T3-L1 Cells

**Figure 2:**
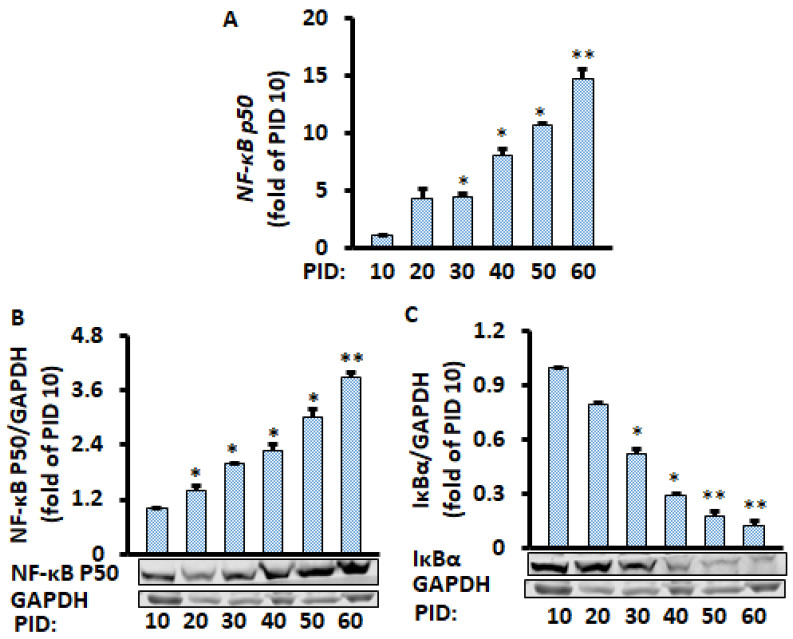
Time-course of changes in gene and protein levels during the 60 days of differentiated adipocyte in conditioned medium. Aging adipocytes chronologically increased NF-κB p50 gene (**A**) and protein (**B**) expression while they decreased IκBα protein level (**C**). Gene and protein levels were measured by RT-PCR and Western blot, respectively, and normalized by GAPDH and EEF2 genes. Data were analyzed using one-way ANOVA, and significant differences were further analyzed using Tukey’s test; * *p* < 0.05, ** *p* < 0.01 compared to PID 10.

Given that the NF-κB signaling pathway plays a central role in chronic inflammation [16,17], we investigated if NF-κB molecules were affected in aging adipocytes. Both protein and mRNA expression of NF-κB p50 were chronologically increased in the aging adipocytes (Figure 2A,B); in contrast, Iκβα protein level was reduced significantly (Figure 2C), suggesting the NF-κB signaling pathway mediates aging-enhanced pro-inflammatory markers. Indeed, reduced Iκβα releases the NF-κB p50/p65 heterodimer in the cytosol and leads to translocation into the nucleus, inducing pro-inflammatory Cytokines [18,19]. The gene expression of NF-κB p65 was also increased with the aging adipocytes (Supplementary Figure S1D), while no significant changes were found in the NF-κB p65 protein expression (Supplementary Figure S1E).

**Figure 3:**
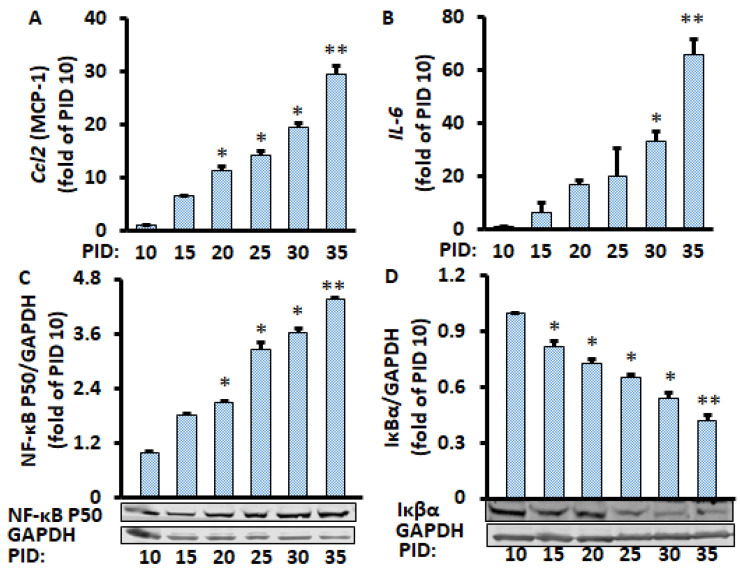
Time course of changes in gene and protein levels during the 35 days of differentiated adipocytes in the unconditioned medium. mRNA of Ccl2 (**A**) and IL-6 (**B**) were measured by RT-PCR, and protein levels of NF-κB p50 (**C**) and IκBα (**D**) were measured by Western blot and normalized by EEF2 and GAPDH, respectively. Data were analyzed using one-way ANOVA, and significant differences were further analyzed using Tukey’s test; * *p* < 0.05, ** *p* < 0.01 compared to PID 10.

Since only half of the medium (1 mL of the 2 mL per well) was replaced every two days during the PID 60 study above, which we defined as a conditioned medium, we wanted to know if the replacement of all of the medium (2 mL per well) every two days during the whole period (defined as the unconditioned medium) would have a similar effect on the production of pro-inflammatory markers from the adipocytes in the conditioned medium. We found that the mRNA levels of pro-inflammatory markers IL-6 and MCP-1 were increased with increasing PID (Figure 3A,B). Similarly, the protein expression of nuclear NF-κB p50 increased over time, while the Iκβα protein expression decreased over time (Figure 3C,D). We also found that lipid accumulation (Supplementary Figure S2) was continuously increased in the cells. These results are very similar to the data when only half of the medium was replaced, indicating that enhanced pro-inflammatory markers in aging adipocytes is independent of the media conditions.

### 2.3. Fasting Glucose Levels Were Increased Chronologically in Mice after 24 Months of Age

**Figure 4:**
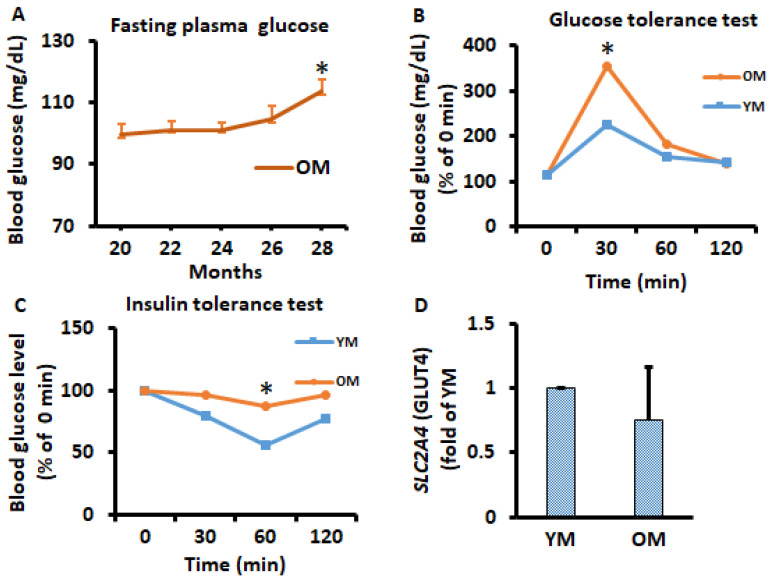
Time course of potential alterations in metabolism during the aging in vivo study. (**A**) Overnight fasting glucose was measured every 2 months in OM. (**B**,**C**) glucose tolerance tests and insulin tolerance tests were performed on month 9 in YM and month 26 of OM by intraperitoneal injection of glucose (2 g/kg body weight) and insulin (1 U/kg body weight. (**D**) SLC2A4 (GLUT4) gene expression was analyzed in EWATs from YM and OM using qPCR. Data were analyzed using one-way ANOVA and *t*-test, and significant differences from one-way ANOVA were further analyzed using Tukey’s test; * *p* < 0.05 compared to 20-month-old mice or YM.

Male 20-month-old C57BL/6 mice were fed a standard chow diet until 37 months of age when 75% of mice died because of aging, and metabolic data were measured and analyzed. Fasting plasma glucose level was measured every two months and, interestingly, fasting glucose levels were increased with advancing time after 24 months of age (Figure 4A). Glucose tolerance tests and insulin tolerance tests were conducted once on young mice (YM) at the age of 9 months and old mice (OM) at the age of 26 months. We found that OM had significantly altered glucose and insulin tolerance (Figure 4B,C). SLC2A4 gene expression was analyzed on EWATs to observe potential metabolic alternation in mice; however, we did not observe significant changes (Figure 4D). SLC2A4 gene expression and pAKT protein expression were also measured periodically on differentiated 3T3-L1 adipocytes; no significant differences were observed (Figure S1G,H).

### 2.4. Pro-Inflammation Markers of the EWATs and Adipocytes from EWATs Were Increased in Extremely Aged Mice

**Figure 5:**
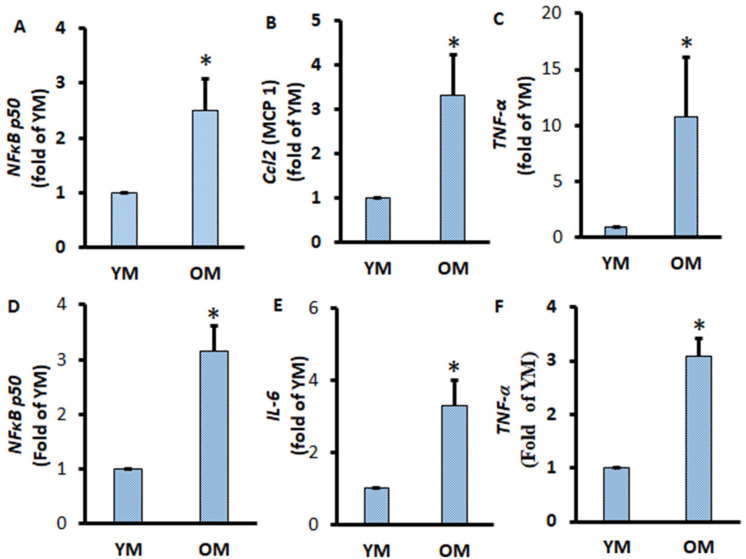
Pro-inflammatory gene expressions of EWATs and adipocytes of aged mice. EWATs were collected from YM and OM mice. The mRNA expressions of NF-κB p50 (**A**), Ccl2 (**B**), and TNF-α (**C**) were measured by RT-qPCR and normalized by GPDH and EEF2. Adipocytes were isolated from EWATs, and the mRNA expressions of NF-κB p50 (**D**), IL-6 (**E**), and TNF-α (**F**) were measured by RT-qPCR and normalized by GAPDH and EEF2. Data were analyzed using *t*-test; * *p* < 0.05 compared to YM.

To further confirm the pro-inflammatory conditions of adipose tissue and adipocytes in the extremely aged mice, we aimed to investigate the pro-inflammatory conditions of EWATs. We found that the pro-inflammatory markers Ccl2 and TNF-α significantly increased in the EWATs of OM compared to YM (Figure 5B,C). Similarly, the gene expression of pro-inflammatory markers IL-6 and TNF-α was significantly increased in adipocytes isolated from OM (Figure 5E,F). The transcription factor NF-κB p50 responsible for the activation of inflammation was also increased significantly in EWATs and adipocytes of OM (Figure 5A,D). These results indicate that aging is associated with increasing pro-inflammation activity in both differentiated adipocytes and in adipocytes from visceral adipose tissues.

## 3. Discussion

Our study demonstrated that the aging of adipocytes enhanced lipid accumulation in conditioned medium until PID 60 and in unconditioned medium until PID 35, which is very similar to the previous report that body fat percentage tends to increase through middle to early old human age (range 40–65 year) in both men and women [20]. We also found that the increased lipid accumulation is accompanied by the enhancement of the pro-inflammatory markers IL-6, TNF-α, and MCP-1 in differentiated 3T3-L1 cells. Interestingly, NFkB p50, a key molecule of transcription factor NF-κB was increased chronologically in the aging of adipocytes in both the conditioned and unconditioned medium. On the contrary, IκΒα, an inhibitor of NF-κB, was significantly decreased in the aging adipocytes. Therefore, chronic accumulated lipid produces more pro-inflammatory markers such as IL-6, TNF-α, and MCP-1, which is associated with the NF-κB pathway. Our in vivo study confirmed that the extreme age visceral adipose tissues and adipocytes was associated with increasing pro-inflammatory cytokines following the NF-κB pathway activation. These results are in line with previous reports that aging enhances adipose tissue pro-inflammatory cytokine expression in visceral adipose tissue [4,5,7]. Wu et al. reported that adipocytes are the principal contributors to pro-inflammation activity in the aging process by regulating the NF-κB pathway [7]. Our in vivo study also confirmed that pro-inflammatory markers were enhanced through the NF-κB pathway as time progressed in the adipose tissue of aged mice. Interestingly, we also found that there was a chronic increase in fasting blood glucose levels after certain ages, indicating that aging progressively increases pro-inflammatory cytokines and might contribute to the development of type 2 diabetes in aged subjects.

The IκB kinase is an enzyme complex, also called the IKK complex, containing IKKα, IKKβ, and IKKγ proteins, which are upstream of the NF-κB signal transduction factors. We found that IKKβ and IKKγ increased over time (Supplementary Figure S1A,B). IKKε is another protein, separate from the IKK complex but similar to IKKα and IKKβ, and plays an essential role in regulating inflammatory responses; overexpression of wild-type IKKε phosphorylates IκΒα, which induces the release of NF-κB [21,22]. Our experiment also demonstrated that IKKε was increased over time in the aging of adipocytes (Supplementary Figure S1C).

3T3-L1 preadipocytes have been extensively used in vitro models for white adipocytes differentiation, derived initially from Swiss mouse embryo tissue (mouse fibroblast line 3T3). Differentiated 3T3-L1 adipocytes have a gene expression profile that is quite similar to white adipocytes; they also possess the characteristics of brown adipocytes in specific microenvironments but do not possess the characteristics of beige adipocytes [23]. We used extremely aged mice fed a standard chow diet in the present study because the aim of this study was to investigate if and how aging adipocytes produce pro-inflammation activity in physiological conditions with minimum external intervention. These healthy aging mice with a standard chow diet may mimic the physiological environment of healthy aged persons (around 65–85 years old). Therefore, this study provides clues to understand why aging enhances chronic inflammation in healthy humans with a regular diet. In the next study, we will use aging obese mice, which may give other explanations of how extreme white fat can accelerate the chronic inflammation in obese subjects, given that over two-thirds of American adults are overweight or obese.

About 1–1.5% of older adipocytes are typically eliminated by apoptosis and replaced by new adipocytes each day in mouse adipose tissue [24]. This means that the adipocyte lifespan is about 60–100 days, which is similar to our aging adipocytes model. A previous study found that IL-6 mRNA increased from PID 5 and reached the maximum at PID 15 [14]. However, our results show that TNF-α and IL-6 levels continuously increased from PID 10 and reached a maximum on PID 60. This disagreement may come from the difference in methods of changing medium, since we only replaced half of the medium, whereas the culture medium was entirely replaced each time in the previous report [14]. Therefore, we designed our second experiment, where the culture medium was completely replaced every 2 days for 35 days. Instead of 60 days, we chose 35 days because the adiponectin and PPAR-γ expression increased at the highest level on day 20–30 (Supplementary Figure S1F,I,J), while inflammatory marker expressions were consistently increased. Therefore, it was not necessary to grow for a longer time to prologue the inflammatory conditions. In addition, these results indicate the complexity of the interactions between adiponectin/PPAR-γ and the inflammatory markers; high levels of adiponectin/PPAR-γ did not mitigate the chronic inflammation in adipocytes, although both adiponectin and PPAR-γ are well-known as anti-inflammatory molecules. This is in line with the previously reported paradox and controversy of the anti-inflammatory effect and mechanism of adiponectin [25] and PPAR-γ [26]. To make sure aged adipocytes had truly gone through aging, we analyzed cleaved caspase-3, a hallmark of aging [27]. Our results showed that cleaved caspase-3 protein expression increased in differentiated adipocytes, and caspase-3 mRNA increased in adipocytes from EWATs (Supplementary Figure S3).

This study aimed to investigate if and how aging adipocytes produce pro-inflammation activity in physiological conditions with minimum external intervention. Therefore, aging adipocytes with the conditioned medium, unconditioned medium, and healthy aging mice fed a standard chow diet were used in this investigation. The conditioned medium (only half of the medium was changed every two days) could mimic the physiological microenvironment to minimize the sharp reduction of chemokines, growth factors, and other active molecules. In fact, compared to normal plasma, the levels of 12 chemokines, 4 growth factors, 4 matrix metalloproteases, and 3 adhesion molecules are lower in heat-inactivated plasma [28], which was added in our cell culture medium. After observing the progressively increased pro-inflammatory markers in aging adipocytes with the conditioned medium, we wanted to assess if changing the total volume of medium (previously called unconditioned medium) would have a similar effect on the production of pro-inflammatory markers. Our results show that the overall patterns of increasing pro-inflammatory markers were the same in the two different media, although there was a slight difference between the two medium conditions. These aging-induced pro-inflammatory markers in adipocytes were also found in extremely aged mice fed a standard chow diet. Therefore, we conclude that aging plays a critical role in chronic pro-inflammatory cytokine production in adipose tissue.

To our knowledge, this study demonstrated for the first time that aging adipocytes increased the production of pro-inflammatory makers chronologically, including TNF-α, IL-6, and MCP-1, which might be mediated by suppressing IκΒα and enhancing the NF-κB pathway. Our model may also challenge the prevailing hypothesis that obesity-associated metabolic decline is not due to adiposity per se, but rather due to the insufficient capacity of adipose tissue to expand further [29]. Our model can be used for therapeutic approaches to prevent inflammation-associated chronic diseases. In conclusion, aging adipocytes chronologically increased pro-inflammatory markers through the NF-κB pathway.

## 4. Materials and Methods

### 4.1. Cell Culture

3T3-L1 preadipocytes (American Type Culture Collection, cat #CL-173, Manassas, VA, USA) were induced to adipocytes as described in [30] and maintained for 35 or 60 days. In brief, 3T3-L1 cells were sown in 6-well plates in a culture medium consisting of high-glucose DMEM (Thermo fisher, cat#11965118) containing 4.5 g L^−1^ glucose, 10% FBS, and 1% P/S. After reaching 100% confluence, a cocktail of 0.5 mM IBMX, 1 μM dexamethasone, 1 μM rosiglitazone, and 10 μg mL^−1^ insulin with culture medium was added to induce adipocyte differentiation for 3 days. On PID 3, cells were fed with 2 mL culture medium with 10 μg mL^−1^ insulin every two days until day PID 10. From PID 10, out of 2 mL culture medium, half of the culture medium (1 mL per well) was replaced every two days until PID 60, which we defined as a conditioned medium; or, the total culture medium (2 mL per well) was replaced every two days until PID 35, which we defined as the unconditioned medium. Media and cell samples were harvested on PID 10, 20, 30, 40, and 60 for conditioned experiments and PID 10, 15, 20, 25, 30, and 35 for unconditioned experiments.

### 4.2. Animal Study

Male C57BL/6 mice (9 months or 20 months old) were purchased from the National Institute on Aging (Bethesda, MD, USA). Mice were housed in a controlled animal facility (23 ± 2 °C; 12-h light/dark cycle) and provided free access to chow diet (Teklad Global Rodent chow diet, Harlan, IN, USA). Nine-month-old YM were euthanized by inhalation of CO_2_ immediately after the one-week acclimation period, and tissue samples were collected. Twenty-month-old OM were provided with tap water and a chow diet for 37 weeks. Bodyweight, food, and water intake were monitored weekly; fasting glucose level was measured every 2 months; insulin and glucose tolerance test were conducted at 9 months in YM and 26 months in OM. The general clinical condition and mortality of the mice were monitored daily. After the 37th week (approximately 29 months of age), 25% of mice survived, and the remaining mice were euthanized by inhalation of CO_2_. Tissue samples, including EWATs, were collected in liquid nitrogen. All the samples were frozen at −80 °C immediately after collection. The animal protocol was approved by the Institutional Animal Care and Use Committee of Tennessee State University, USA (Assurance code # 15-11-637).

### 4.3. Adipocytes Isolation

EWATs were collected under sterile conditions and put in a dish containing 1 mL of sterile PBS with 2% BSA. Adipose tissue was minced into pieces of approximately 5–10 mg and incubated in 2 mg/mL collagenase type I (Worthington Biochemical, Lakewood, NJ, USA) in a shaking water bath at 37 °C for 1.5 h. Digested cells were filtered through a 250-μm mesh and then centrifuged at 450× *g* for 10 min. The cells floating on the top were transferred to a new tube as adipocytes. Adipocytes were washed twice with DMEM plus 10% FBS before the experiments were conducted.

### 4.4. Oil Red O Staining

For lipid accumulation, the differentiated adipocytes were stained with 60% Oil Red O solution (0.5 mL per well) for 15 min at room temperature and then washed twice with distilled water. Photographs were taken under an inverted microscope. Accumulated lipids were dissolved in 0.5 mL 100% isopropanol and measured using a Synergy H1 microplate reader (Bio-Tek Instruments, Inc., Winooski, VT, USA) at 490 nm as previously described [30].

### 4.5. ELISA

Soluble pro-inflammatory markers IL-6 (mouse cat#ADI-900-045) and TNF-α (mouse cat#ADI-900-047) were measured from the differentiated cells using ELISA kits (Enzo Life Sciences, Farmingdale, NY, USA) according to the manufacturer’s instructions.

### 4.6. Western Blot

Protein levels of IκBα (cat#9242), adiponectin (cat#2789), NF-κB p65 (cat#8242), cleaved caspase-3 (cat#9661), and NF-κB p50 (cat#3035) were measured by Western blots as previously described [30]. Primary antibodies of target proteins were from Cell Signaling Technology (Beverly, MA, USA). The target protein levels were normalized by GAPDH (cat#5174), and all antibodies were diluted in a 1:1000 ratio.

### 4.7. qPCR

The mRNA expression of pro-inflammatory markers and molecules of the NF-κB pathway was evaluated by RT-qPCR, as described before [31]. The target mRNA levels were normalized by GAPDH and EEF2. Primers GAPDH, EEF2, NF-κB p65, NF-κB p50, IL-6, IKKβ, IKKγ, IKKε, Ccl2 (MCP-1), IL-1β, SLC2A4 (GLUT4), and TNF-α were synthesized by Integrated DNA Technologies (Coralville, IA, USA). Sequences of primers are provided in Supplementary Table S1.

### 4.8. Statistics

All values are presented as mean ± SEM of at least three independent experiments. SPSS software was used to analyze all data with *t*-test or one-way ANOVA, and significant differences between treatment groups further analyzed using Tukey’s test. Differences with *p* < 0.05 were considered significant.

## Data Availability

Data is contained within the article or supplementary material.

## Supplementary Materials

The following are available online at https://www.mdpi.com/article/10.3390/metabo11050292/s1, Figure S1: Aging of adipocytes upregulated mRNA IKKβ (A), IKKγ (B), IKKε (C), and NF-κB p65 (D) in conditioned media microenvironment during PID 60, while protein adiponectin (F) and PPAR-γ (I) and mRNA SLC2A4 (H) were upregulated at a certain point, Figure S2: Time course of changes in lipid levels during PID 35 of differentiated 3T3-L1 adipocytes in the unconditioned medium, Figure S3: (A) Time course of cleaved caspase-3 protein expression during the 60 days in the conditioned differentiated 3T3-L1 adipocytes; (B) caspase-3 mRNA expression of adipocytes from YM and OM, Table S1: Sequences of primers.
