# Supplementary material for: The Aging of Adipocytes Increases Expression of Pro-Inflammatory Cytokines Chronologically
Source: Metabolites. 2021 May 1;11(5):292. doi: 10.3390/metabo11050292 (PMC8147339; doi:10.3390/metabo11050292)
Supplement: Supplementary file 1 [file metabolites-11-00292-s001.zip › metabolites-1141739-supplementary.pdf]

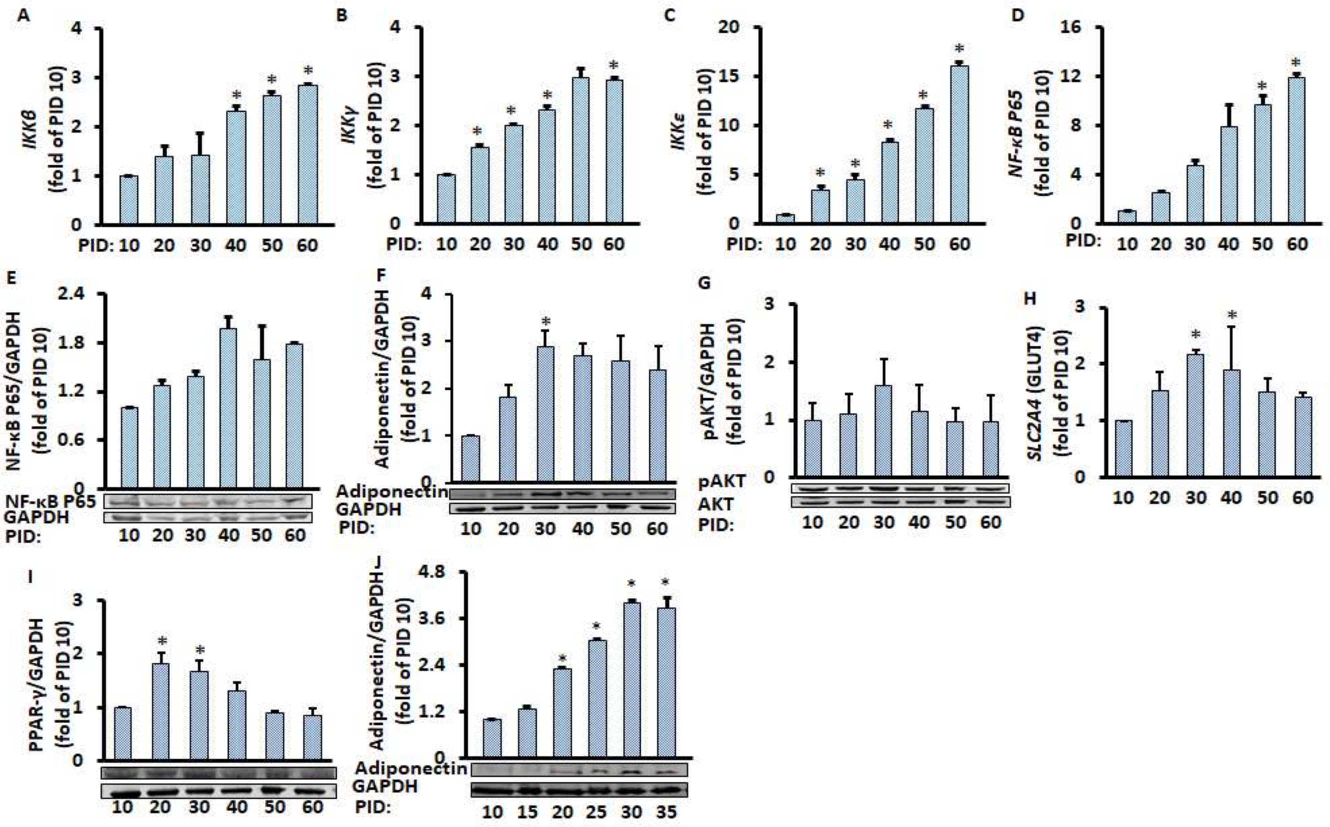

Figure S1: Aging of adipocytes upregulated mRNA of IKKβ (**A**), IKKγ (**B**), IKKε (**C**), and NF-κB p65 (**D**) in conditioned media microenvironment during PID 60, while protein adiponectin (**F**) and PPAR-γ (**I**), and mRNA SLC2A4 (**H**) were upregulated at a certain point. No changes were found in protein NF-κB p65 (**E**) and pAKT (**G**) expression during PID 60 in conditioned media. Protein expression of adiponectin (**J**) in unconditioned medium also increased gradually at a certain level during PID 35. Gene and protein were measured by RT-PCR and Western blot, respectively, and normalized by GAPDH. Data were analyzed using one-way ANOVA, and significant differences were further analyzed using Tukey's test; * $p < 0.05$ compared to PID 10.

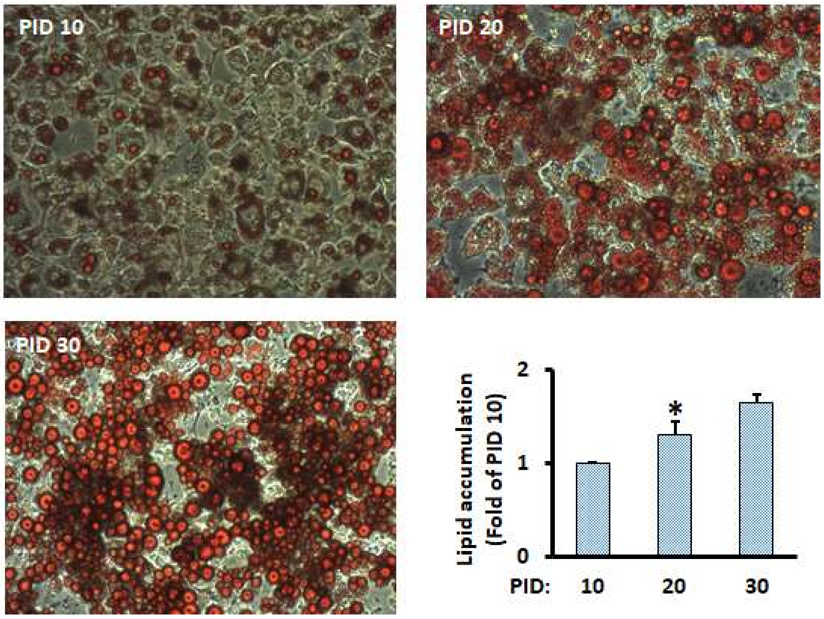

Figure 2. Time course of changes in lipid levels during PID 35 of differentiated 3T3-L1 adipocytes in unconditioned medium. Representative images were taken after Oil Red O staining, and lipids were measured using a spectrophotometer, and a bar graph is shown. Data were analyzed using one-way ANOVA, and significant differences were further analyzed using Tukey's test; $*p < 0.05$ compared to PID 10.

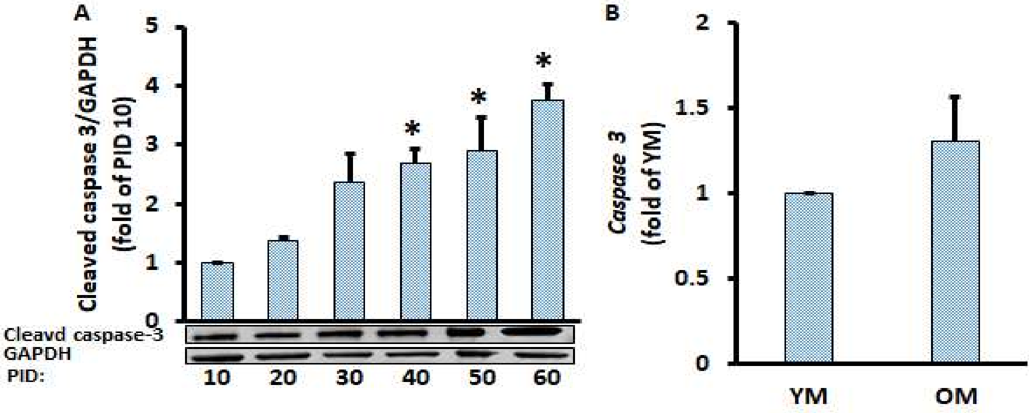

Figure 3. (**A**) Time course of cleaved caspase-3 protein expression during the 60 days in the conditioned differentiated 3T3-L1 adipocytes; (**B**) caspase-3 mRNA expression of adipocytes from YM and OM. Gene and protein were measured by RT-PCR and Western blot, respectively, and normalized by GAPDH and EEF2 genes. Data were analyzed using one-way ANOVA and *t*-test, and significant differences from one-way ANOVA were further analyzed using Tukey's test; *$p < 0.05$, compared to PID 10 or YM.

Table 1. Sequences of primers.

Genes		Sequence (5′-3′)
IKKε	Forward	GGGAGAGTCTTTGCCTGATT
	Reverse	CATCAGTATGCC ACA GGTAGTT
IKKβ	Forward	GCCAAGCTGGACTTCTTCA
	Reverse	CCAAGCCAGCAGCAATTTATC
IKKγ	Forward	CAAGACTACGACAGCCACATTA
	Reverse	CAGCCTCCTCTTTCAGCTTATC
NFKB p50	Forward	GGATGA CAGAGGCGTGTATTAG
	Reverse	CCTTCTCTCTGTCTGTGAGTTG
NFKB p65	Forward	GCTCAAGATCTGCCGAGTAAA
	Reverse	GTCCCGTGAAATACACCTCAA
TNF-α	Forward	CTG AGT TCT GCA AAG GGA GAG
	Reverse	CCT CAG GGA AGA ATC TGG AAA G
Casp3	Forward	CATAAGAGCACTGGAATGTC
	Reverse	GCTCCTTTTGCTATGATCTTC
Ccl2 (Mcp1)	Forward	AGTAGGCTGGAGAGCTACAA
	Reverse	GTATGTCTGGACCCATTCCTTC
IL6	Forward	AGTTGTGCAATGGCAATTCTG
	Reverse	TCCAGTTTGGTAGCATCCATC
GAPDH	Forward	CTG AAG CCA GCT CTC TCT TCC T
	Reverse	GCTCCTTTTGCTATGATCTTC
Eef2	Forward	AGAACATATATTGCTGGCG
	Reverse	CAACAGGGTTAGATTTCTTG
SLC2A4	Forward	CCAGAGACCACCTACATGGC
	Reverse	GTCGGCATGGGTTTCCAGAAT
